# Supplementary material for: The E3 ubiquitin ligase mindbomb1 controls planar cell polarity-dependent convergent extension movements during zebrafish gastrulation
Source: eLife. 2022 Feb 10;11:e71928. doi: 10.7554/eLife.71928 (PMC8937233; doi:10.7554/eLife.71928)
Supplement: Figure 1—source data 1. [file elife-71928-fig1-data1.docx]

**Figure 1-source data 1: Complete statistical information for the experiments reported in Figure 1 and Figure 1-figure supplement 1**

**Figure 1A: Axis extension angle in mib1 morphants**

|  | *Mean value* | *Standard deviation* | *Number of embryos* |
| --- | --- | --- | --- |
| WT | 198.8 | 8.9 | 53 |
| MO mib1 | 185.7 | 12.1 | 54 |
| MO mib1 + RNA WT mib1 | 198.5 | 9.7 | 43 |
|  | | | |
| *Test statistics for Kruskal-Wallis test* | | | |
| F = 38.7 | p = 3.9E-09 |  |  |
|  | | | |
| *Adjusted p-values for pairwise comparisons (Dunn post-hoc test, Holm correction)* | | | |
|  | MO mib1 | MO mib1 + RNA WT mib1 |  |
| WT | 5.3E-08 | 0.72 |  |
| MO mib1 |  | 1.32E-06 |  |

**Figure 1B: Notochord, somite & neural plate width in WT and mib1 morphants**

|  | *Mean notochord width* | *Standard deviation* | *Number of embryos* |
| --- | --- | --- | --- |
| WT | 24.7 | 3.5 | 40 |
| MO mib1 | 32.2 | 5.0 | 39 |
|  | | | |
| *Welch’s t-test:* | | | |
| p = 8.2E-11 |  |  |  |

|  | *Mean somite width* | *Standard deviation* | *Number of embryos* |
| --- | --- | --- | --- |
| WT | 249 | 26.2 | 40 |
| MO mib1 | 297 | 39.9 | 39 |
|  | | | |
| *Welch’s t-test:* | | | |
| p = 2.3E-08 |  |  |  |

|  | *Mean neural plate width* | *Standard deviation* | *Number of embryos* |
| --- | --- | --- | --- |
| WT | 299 | 37.1 | 40 |
| MO mib1 | 376 | 55.6 | 39 |
|  | | | |
| *Welch’s t-test:* | | | |
| p = 5.4E-10 |  |  |  |

**Figure 1D: Axis extension angle in *mib1^ta52b^***

|  | *Mean value* | *Standard deviation* | *Number of embryos* |
| --- | --- | --- | --- |
| *mib1[+/+]* | 199.9 | 11.6 | 29 |
| *mib1[ta52b/+]* | 203.5 | 10.6 | 62 |
| *mib1[ta52b/ta52b]* | 202.8 | 11.5 | 48 |
|  | | | |
| *Test statistics for One Way Anova* | | | |
| F = 1.1 | p = 0.34 |  |  |

**Figure 1E: Axis extension angle in mib1 morphants injected with NICD RNA**

|  | *Mean value* | *Standard deviation* | *Number of embryos* |
| --- | --- | --- | --- |
| WT | 201.0 | 7.8 | 47 |
| MO mib1 | 189.1 | 10.2 | 57 |
| MO mib1 + RNA NICD | 189.3 | 12.8 | 83 |
| RNA NICD | 197.0 | 8.8 | 41 |
|  | | | |
| *Test statistics for Welch’s Anova* | | | |
| F = 18.4 | p = 7.4E-10 |  |  |
|  | | | |
| *Adjusted p-values for pairwise comparisons (Games Howell Test)* | | | |
|  | MO mib1 | MO mib1 + RNA NICD | RNA NICD |
| WT | 8.6E-08 | 2.0E-07 | 0.34 |
| MO mib1 |  | 0.99 | 5.2E-04 |
| MO mib1 + RNA NICD |  |  | 1.0E-03 |

**Figure 1F: Axis extension angle in mib1 morphants injected with mib1[ta52b] RNA**

|  | *Mean value* | *Standard deviation* | *Number of embryos* |
| --- | --- | --- | --- |
| WT | 201.3 | 8.1 | 38 |
| MO mib1 | 188.6 | 9.7 | 40 |
| MO mib1 + RNA mib1[ta52b] | 199.8 | 10.1 | 38 |
| RNA mib1[ta52b] | 202.7 | 9.3 | 20 |
|  | | | |
| *Test statistics for One way Anova* | | | |
| F = 17.0 | p = 2.2E-09 |  |  |
|  | | | |
| *Adjusted p-values for pairwise comparisons (Tukey HSD test)* | | | |
|  | MO mib1 | MO mib1 + RNA mib1[ta52b] | RNA mib1[ta52b] |
| WT | 1.1E-07 | 0.90 | 0.95 |
| MO mib1 |  | 2.8E-06 | 1.1E-06 |
| MO mib1 + RNA mib1[ta52b] |  |  | 0.69 |

**Figure 1G: Axis extension angle in *mib1^tfi91^***

|  | *Mean value* | *Standard deviation* | *Number of embryos* |
| --- | --- | --- | --- |
| WT siblings | 197.9 | 11.0 | 175 |
| *mib1[tfi91/tfi91]* | 192.4 | 11.4 | 67 |
|  | | | |
| *Wilcoxon test:* | | | |
| p = 1.9E-03 |  |  |  |

**Figure 1G: Axis extension angle in *mib1^tfi91/nce2a^***

|  | *Mean value* | *Standard deviation* | *Number of embryos* |
| --- | --- | --- | --- |
| WT siblings | 202.9 | 10.4 | 55 |
| *mib1[tfi91/nce2a]* | 195.8 | 11.5 | 10 |
|  | | | |
| *t-test:* | | | |
| p = 1.6E-02 |  |  |  |

**Figure 1G: Axis extension angle in *mib1^nce2a^***

|  | *Mean value* | *Standard deviation* | *Number of embryos* |
| --- | --- | --- | --- |
| WT siblings | 210.9 | 10.0 | 114 |
| *mib1[nce2a/nce2a]* | 207.4 | 9.5 | 50 |
|  | | | |
| *Welch’s t-test:* | | | |
| p = 3.9E-02 |  |  |  |

**Figure 1-figure supplement 1A: *shhb* axis extension angle in MO mib1, Mib1ΔRF123 and Mib1ΔRF3**

|  | *Mean value* | *Standard deviation* | *Number of embryos* |
| --- | --- | --- | --- |
| WT | 165.8 | 9.2 | 58 |
| MO mib1 | 141.2 | 11.8 | 40 |
| Mib1ΔRF123 | 149.4 | 10.6 | 39 |
| Mib1ΔRF3 | 146.7 | 12.4 | 39 |
|  | | | |
| *Test statistics for One way Anova* | | | |
| F = 47.8 | p < 2.2E-16 |  |  |
|  | | | |
| *Adjusted p-values for pairwise comparisons (Tukey HSD Test)* | | | |
|  | MO mib1 | Mib1ΔRF123 | Mib1ΔRF3 |
| WT | 3.0E-14 | 8.1E-11 | 1.2E-13 |
| MO mib1 |  | 5.3E-03 | 0.11 |
| Mib1ΔRF123 |  |  | 0.70 |

**Figure 1-figure supplement 1B: *foxA3* axis extension angle in MO mib1, Mib1ΔRF123 and Mib1ΔRF3**

|  | *Mean value* | *Standard deviation* | *Number of embryos* |
| --- | --- | --- | --- |
| WT | 180.3 | 7.4 | 74 |
| MO mib1 | 160.6 | 10.3 | 37 |
| Mib1ΔRF123 | 173.1 | 9.7 | 39 |
| Mib1ΔRF3 | 170.0 | 9.5 | 37 |
|  | | | |
| *Test statistics for Kruskall-Wallis test:* | | | |
| Chi-squared = 69.0 | p = 7.0E-15 |  |  |
|  | | | |
| *Adjusted p-values for pairwise comparisons (Dunn post-hoc test, Holm correction)* | | | |
|  | MO mib1 | Mib1ΔRF123 | Mib1ΔRF3 |
| WT | 5.3E-15 | 3.2E-03 | 1.8E-05 |
| MO mib1 |  | 9.1E-05 | 6.4E-03 |
| Mib1ΔRF123 |  |  | 0.21 |

**Figure 1-figure supplement 1C: *foxA3* notochord width in MO mib1, Mib1ΔRF123 and Mib1ΔRF3**

|  | *Mean value* | *Standard deviation* | *Number of embryos* |
| --- | --- | --- | --- |
| WT | 52.7 | 6.5 | 82 |
| MO mib1 | 61.2 | 9.0 | 38 |
| Mib1ΔRF123 | 60.3 | 7.3 | 39 |
| Mib1ΔRF3 | 59.4 | 7.9 | 36 |
|  | | | |
| *Test statistics for One way Anova* | | | |
| F = 16.8 | p = 9.5E-10 |  |  |
|  | | | |
| *Adjusted p-values for pairwise comparisons (Tukey HSD Test)* | | | |
|  | MO mib1 | Mib1ΔRF123 | Mib1ΔRF3 |
| WT | 1.6E-07 | 2.5E-06 | 7.5E-05 |
| MO mib1 |  | 0.95 | 0.72 |
| Mib1ΔRF123 |  |  | 0.95 |
