# Supplementary material for: The E3 ubiquitin ligase mindbomb1 controls planar cell polarity-dependent convergent extension movements during zebrafish gastrulation
Source: eLife. 2022 Feb 10;11:e71928. doi: 10.7554/eLife.71928 (PMC8937233; doi:10.7554/eLife.71928)
Supplement: Figure 2—source data 1. [file elife-71928-fig2-data1.docx]

**Figure 2-source data 1: Complete statistical information for the experiments reported in Figure 2**

**Figure 2A: Axis extension angle in mib1 morphants injected with RhoA RNA**

|  | *Mean value* | *Standard deviation* | *Number of embryos* |
| --- | --- | --- | --- |
| WT | 200.3 | 8.5 | 30 |
| MO mib1 | 180.1 | 8.6 | 31 |
| MO mib1 + RNA RhoA | 197.9 | 9.9 | 38 |
| RNA RhoA | 203.0 | 10.7 | 33 |
|  | | | |
| *Test statistics for One way Anova* | | | |
| F = 37.2 | p = 2.3E-17 |  |  |
|  | | | |
| *Adjusted p-values for pairwise comparisons (Tukey HSD test)* | | | |
|  | MO mib1 | MO mib1 + RNA RhoA | RNA RhoA |
| WT | 8.2E-13 | 0.74 | 0.68 |
| MO mib1 |  | 1.5E-11 | 3.0E-14 |
| MO mib1 + RNA RhoA |  |  | 0.12 |

**Figure 2B: Axis extension angle in mib1 morphants injected with Mib1-ΔRF123 RNA**

|  | *Mean value* | *Standard deviation* | *Number of embryos* |
| --- | --- | --- | --- |
| WT | 206.4 | 9.9 | 84 |
| MO mib1 | 192.5 | 8.6 | 68 |
| MO mib1 + RNA Mib1-ΔRF123 | 186.7 | 9.5 | 111 |
| RNA Mib1-ΔRF123 | 193.8 | 8.2 | 84 |
|  | | | |
| *Test statistics for One wayAnova* | | | |
| F = 76.6 | p = 6.4E-38 |  |  |
|  | | | |
| *Adjusted p-values for pairwise comparisons (Tukey HSD Test)* | | | |
|  | MO mib1 | MO mib1 + RNA Mib1-ΔRF123 | RNA Mib1-ΔRF123 |
| WT | <2.2E-16 | <2.2E-16 | <2.2E-16 |
| MO mib1 |  | 8.7E-07 | 0.83 |
| MO mib1 + RNA Mib1-ΔRF123 |  |  | 2.5E-04 |

**Figure 2C: Axis extension angle in mib1 morphants injected with Mib1-ΔRF3 RNA**

|  | *Mean value* | *Standard deviation* | *Number of embryos* |
| --- | --- | --- | --- |
| WT | 202.3 | 9.1 | 45 |
| MO mib1 | 189.4 | 7.8 | 41 |
| MO mib1 + RNA Mib1-ΔRF3 | 180.9 | 13.8 | 48 |
| RNA Mib1-ΔRF3 | 187.2 | 11.9 | 44 |
|  | | | |
| *Test statistics for Welch’s Anova* | | | |
| F = 32.9 | p = 1.1E-14 |  |  |
|  | | | |
| *Adjusted p-values for pairwise comparisons (Games Howell Test)* | | | |
|  | MO mib1 | MO mib1 + RNA Mib1-ΔRF3 | RNA Mib1-ΔRF3 |
| WT | 2.4E-09 | 5.7E-11 | 1.6E-08 |
| MO mib1 |  | 3.1E-03 | 0.75 |
| MO mib1 + RNA Mib1-ΔRF3 |  |  | 0.09 |
