# Supplementary material for: The E3 ubiquitin ligase mindbomb1 controls planar cell polarity-dependent convergent extension movements during zebrafish gastrulation
Source: eLife. 2022 Feb 10;11:e71928. doi: 10.7554/eLife.71928 (PMC8937233; doi:10.7554/eLife.71928)
Supplement: Figure 3—source data 1. [file elife-71928-fig3-data1.docx]

**Figure 3-source data 1: Complete statistical information for the experiments reported in Figure 3 and Figure 3-figure supplement 5**

**Figure 3E: Number of Ryk endosomes in mib1 morphants injected with 3 pg Ryk-GFP RNA**

|  | *Mean value* | *Standard error* | *Sample size* |
| --- | --- | --- | --- |
| WT | 1.94 | 0.22 | 11 embryos – 20 cells/embryo |
| MO mib1 | 0.64 | 0.09 | 15 embryos – 20 cells/embryo |
|  | | | |
| *t-test:* | | | |
| p = 4.1E-06 |  |  |  |

**Figure 3E: Ryk endosomes in MO mib1 + Mib1-ΔRF123 injected with 12 pg Ryk-GFP RNA**

|  | *Mean value* | *Standard error* | *Number of embryos* |
| --- | --- | --- | --- |
| WT | 4.84 | 0.18 | 27 embryos 20 cells/embryo |
| MO mib1 | 2.21 | 0.19 | 12 embryos 20 cells/embryo |
| MO mib1 + RNA Mib1-ΔRF123 | 0.88 | 0.13 | 15 embryos 20 cells/embryo |
|  | | | |
| *Test statistics for Kruskal-Wallis test* | | | |
| Chi-squared = 43.5 | p = 3.5E-10 |  |  |
|  | | | |
| *Adjusted p-values for pairwise comparisons (Dunn post-hoc test, Holm correction)* | | | |
|  | MO mib1 | MO mib1 + RNA Mib1-ΔRF123 |  |
| WT | 3.7E-04 | 5.7E-10 |  |
| MO mib1 |  | 0.05 |  |

**Figure 3J: Number of Ryk endosomes in *mib1^tfi91^* mutants injected with 12 pg Ryk-GFP RNA**

|  | *Mean value* | *Standard error* | *Sample size* |
| --- | --- | --- | --- |
| *mib1[+/+]* | 4.58 | 0.19 | 12 embryos – 20 cells/embryo |
| *mib1[tfi91/tfi91]* | 2.30 | 0.22 | 14 embryos – 20 cells/embryo |
|  | | | |
| *t-test:* | | | |
| p = 4.8E-08 |  |  |  |

**Figure 3K: Axis extension angle in mib1 morphants injected with Ryk-GFP**

|  | *Mean value* | *Standard deviation* | *Number of embryos* |
| --- | --- | --- | --- |
| WT | 212.9 | 10.0 | 61 |
| MO mib1 | 201.2 | 8.4 | 65 |
| MO mib1 + RNA Ryk-GFP | 211.4 | 9.8 | 82 |
| MO mib1 + RNA Mib1-ΔRF123 + RNA Ryk-GFP | 190.6 | 8.6 | 94 |
|  | | | |
| *Test statistics for Kruskal-Wallis test* | | | |
| Chi-squared = 160 | p = 1.7E-34 |  |  |
|  | | | |
| *Adjusted p-values for pairwise comparisons (Dunn post-hoc test, Holm correction)* | | | |
|  | MO mib1 | MO mib1 + RNA Ryk-GFP | MO mib1 + RNA Mib1-ΔRF123 + RNA Ryk-GFP |
| WT | 1.2E-06 | 0.56 | 4.5E-25 |
| MO mib1 |  | 2.5E-06 | 1.4E-06 |
| MO mib1 + RNA Ryk-GFP |  |  | 2.7E-27 |

**Figure 3L: Axis extension angle in *mib1^tfi91^* mutants injected with Ryk-GFP**

|  | *Mean value* | *Standard deviation* | *Number of embryos* |
| --- | --- | --- | --- |
| *mib1[WT siblings]* | 193.9 | 12.6 | 94 |
| *mib1[tfi91/tfi91]* | 185.9 | 11.9 | 34 |
| *mib1[WT siblings]* +  RNA Ryk-GFP | 192.7 | 10.2 | 80 |
| *mib1[tfi91/tfi91]* + RNA Ryk-GFP | 196.3 | 10.6 | 24 |
|  | | | |
| *Test statistics for One way Anova* | | | |
| F = 5.1 | p = 1.9E-03 |  |  |
|  | | | |
| *Adjusted p-values for pairwise comparisons (Tukey HSD Test)* | | | |
|  | *mib1[tfi91/tfi91]* | *mib1[WT siblings]* +  RNA Ryk-GFP | *mib1[tfi91/tfi91]* +  RNA Ryk-GFP |
| *mib1[WT siblings]* | 3.0E-03 | 0.89 | 0.82 |
| *mib1[tfi91/tfi91]* |  | 0.02 | 4.6E-03 |
| *mib1[WT siblings]* +  RNA Ryk-GFP |  |  | 0.55 |

**Figure 3M: Axis extension angle in mib1 ryk double morphants**

|  | *Mean value* | *Standard deviation* | *Number of embryos* |
| --- | --- | --- | --- |
| WT | 204.5 | 8.7 | 44 |
| MO mib1 | 197.2 | 6.7 | 44 |
| MO ryk | 196.0 | 7.5 | 41 |
| MO mib1 + MO ryk | 183.7 | 8.4 | 78 |
|  | | | |
| *Test statistics for One way Anova* | | | |
| F = 32.9 | p = 1.1E-14 |  |  |
|  | | | |
| *Adjusted p-values for pairwise comparisons (Tukey HSD Test)* | | | |
|  | MO mib1 | MO ryk | MO mib1 + MO ryk |
| WT | 1.6E-04 | 1.1E-05 | <2.2E-16 |
| MO mib1 |  | 0.90 | <2.2E-16 |
| MO ryk |  |  | 5E-13 |

**Figure 3-figure supplement 5: Axis extension angle in mib1 morphants injected with Vangl2 RNA**

|  | *Mean value* | | *Standard deviation* | | *Number of embryos* |
| --- | --- | --- | --- | --- | --- |
| WT | 208.2 | | 7.3 | | 20 |
| MO mib1 | 191.1 | | 6.7 | | 20 |
| MO mib1 + RNA Vangl2 | 190.3 | | 5.8 | | 25 |
| RNA Vangl2 | 203.3 | | 6.1 | | 20 |
|  | | | | | |
| *Test statistics for Kruskall-Wallis test:* | | | | | |
| Chi-squared = 51.6 | | p = 3.7E-11 | |  |  |
|  | | | | | |
| *Adjusted p-values for pairwise comparisons (Dunn post-hoc test, Holm correction)* | | | | | |
|  | | MO mib1 | | MO mib1 + RNA Vangl2 | RNA Vangl2 |
| WT | | 6.9E-07 | | 1.4E-08 | 0.40 |
| MO mib1 | |  | | 0.68 | 1.9E-04 |
| MO mib1 + RNA Vangl2 | |  | |  | 1.5E-05 |
