# Supplementary material for: The E3 ubiquitin ligase mindbomb1 controls planar cell polarity-dependent convergent extension movements during zebrafish gastrulation
Source: eLife. 2022 Feb 10;11:e71928. doi: 10.7554/eLife.71928 (PMC8937233; doi:10.7554/eLife.71928)
Supplement: Figure 4—source data 1. [file elife-71928-fig4-data1.docx]

**Figure 4-source data 1: Complete statistical information for the experiments reported in Figure 4 and Figure 4-figure supplement 1**

**Figure 4D: Axis extension angle in MO ryk + RNA ryk[WT]**

|  | *Mean value* | *Standard deviation* | *Number of embryos* |
| --- | --- | --- | --- |
| WT | 189.7 | 7.8 | 60 |
| MO ryk | 183.2 | 9.0 | 60 |
| MO ryk + RNA ryk[WT] | 188.1 | 7.4 | 60 |
|  | | | |
| *Test statistics for One way Anova:* | | | |
| F = 10.2 | p = 6.7E-05 |  |  |
|  | | | |
| *Adjusted p-values for pairwise comparisons (Dunn post-hoc test, Holm correction)* | | | |
|  | MO ryk | MO ryk + RNA ryk[WT] |  |
| WT | 7.6E-05 | 0.54 |  |
| MO ryk |  | 3.7E-03 |  |

**Figure 4E: Axis extension angle in MO ryk + RNA ryk[nce4g]**

|  | *Mean value* | *Standard deviation* | *Number of embryos* |
| --- | --- | --- | --- |
| WT | 194.0 | 6.8 | 49 |
| MO ryk | 185.0 | 7.8 | 49 |
| MO ryk + RNA ryk[nce4g] | 185.0 | 10.1 | 49 |
|  | | | |
| *Test statistics for Kruskall-Wallis test:* | | | |
| Chi-squared = 33.5 | p = 5.2E-08 |  |  |
|  | | | |
| *Adjusted p-values for pairwise comparisons (Dunn post-hoc test, Holm correction)* | | | |
|  | MO ryk | MO ryk + RNA ryk[nce4g] |  |
| WT | 1.7E-06 | 1.0E-06 |  |
| MO ryk |  | 0.86 |  |

**Figure 4G: Axis extension angle in Zygotic *ryk^nce4g^* mutants**

|  | *Mean value* | *Standard deviation* | *Number of embryos* |
| --- | --- | --- | --- |
| *ryk[+/+]* | 189.1 | 7.3 | 49 |
| *ryk[nce4g/+]* | 189.4 | 7.9 | 71 |
| *Z ryk[nce4g/nce4g]* | 188.7 | 8.4 | 35 |
|  | | | |
| *Test statistics for One Way Anova* | | | |
| F = 0.09 | p = 0.91 |  |  |

**Figure 4H: *shhb* axis extension angle in MZ *ryk^nce4g^*mutants**

|  | *Mean value* | *Standard deviation* | *Sample size* |
| --- | --- | --- | --- |
| MZ *ryk[+/+]* | 164.2 | 10.2 | 57 |
| MZ *ryk[vms4g/vms4g]* | 146.2 | 13.1 | 58 |
|  | | | |
| *Welch’s t-test:* | | | |
| p = 4.6E-13 |  |  |  |

**Figure 4I: *foxA3* notochord width in MZ *ryk^nce4g^* mutants**

|  | *Mean value* | *Standard deviation* | *Sample size* |
| --- | --- | --- | --- |
| MZ *ryk[+/+]* | 43.6 | 4.9 | 36 |
| MZ *ryk[vms4g/vms4g]* | 50.8 | 7.2 | 38 |
|  | | | |
| *t-test:* | | | |
| p = 3.3E-06 |  |  |  |

**Figure 4J: Axis extension angle in Maternal Zygotic *ryk^nce4g^* + ryk RNA**

|  | *Mean value* | *Standard deviation* | *Number of embryos* |
| --- | --- | --- | --- |
| MZ *ryk[+/+]* | 194.4 | 9.5 | 125 |
| MZ *ryk[nce4g/nce4g]* | 181.8 | 8.8 | 102 |
| MZ *ryk[nce4g/nce4g]*  + RNA ryk (0.75 pg) | 184.8 | 6.3 | 50 |
| MZ *ryk[nce4g/nce4g]*  + RNA ryk (1.5 pg) | 189.2 | 7.3 | 50 |
|  | | | |
| *Test statistics for Welch’s Anova* | | | |
| F = 39.8 | p = 6.9E-19 |  |  |
|  | | | |
| *Adjusted p-values for pairwise comparisons (Tukey HSD Test)* | | | |
|  | MZ *ryk[nce4g/nce4g]* | MZ *ryk[nce4g/nce4g]*  + RNA ryk (0.75 pg) | MZ *ryk[nce4g/nce4g]* + RNA ryk (1.5 pg) |
| MZ *ryk[+/+]* | 1.9E-14 | 1.1E-11 | 9.8E-04 |
| MZ *ryk[nce4g/nce4g]* |  | 0.08 | 1.7E-06 |
| MZ *ryk[nce4g/nce4g]* +  RNA ryk (0.75 pg) |  |  | 9.8E-03 |

**Figure 4K: Axis extension angle in *ryk ; mib1* double mutants**

|  | *Mean value* | *Standard deviation* | *Sample size* |
| --- | --- | --- | --- |
| MZ *ryk[vms4g/vms4g] ; mib1[WT sibs]* | 181.4 | 9.1 | 151 |
| MZ *ryk[vms4g/vms4g] ; mib1[tfi91/tfi91]* | 182.7 | 10.3 | 64 |
|  | | | |
| *t-test:* | | | |
| p = 0.36 |  |  |  |

**Figure 4-figure supplement 1E: *foxA3* axis extension angle in MZ *ryk^nce4g^***

|  | *Mean value* | *Standard deviation* | *Sample size* |
| --- | --- | --- | --- |
| MZ *ryk[+/+]* | 177.3 | 5.0 | 36 |
| MZ *ryk[vms4g/vms4g]* | 165.2 | 8.1 | 38 |
|  | | | |
| *Welch’s t-test:* | | | |
| p = 9.2E-11 |  |  |  |

**Figure 4-figure supplement 1I: Axis extension angle in Maternal Zygotic *ryk^nce4g^* + MO ryk**

|  | *Mean value* | *Standard deviation* | *Sample size* |
| --- | --- | --- | --- |
| MZ *ryk[vms4g/vms4g]* | 179.8 | 6.6 | 30 |
| MZ *ryk[vms4g/vms4g] +* MO ryk | 181.5 | 5.4 | 30 |
|  | | | |
| *Wilcoxon test:* | | | |
| p = 0.37 |  |  |  |
