# Supplementary material for: The E3 ubiquitin ligase mindbomb1 controls planar cell polarity-dependent convergent extension movements during zebrafish gastrulation
Source: eLife. 2022 Feb 10;11:e71928. doi: 10.7554/eLife.71928 (PMC8937233; doi:10.7554/eLife.71928)
Supplement: Figure 5—source data 1. [file elife-71928-fig5-data1.docx]

**Figure 5-source data 1: Complete statistical information for the experiments reported in Figure 5**

**Figure 5A: Axis extension angle in mib1 wnt5b double morphants**

|  | *Mean value* | *Standard deviation* | *Number of embryos* |
| --- | --- | --- | --- |
| WT | 201.1 | 7.5 | 60 |
| MO mib1 | 194.1 | 10.0 | 60 |
| MO wnt5b | 199.6 | 7.1 | 58 |
| MO mib1 + MO wnt5b | 188.0 | 6.6 | 60 |
|  | | | |
| *Test statistics for One way Anova* | | | |
| F = 42.7 | p < 2.2E-16 |  |  |
|  | | | |
| *Adjusted p-values for pairwise comparisons (Tukey HSD Test)* | | | |
|  | MO mib1 | MO wnt5b | MO mib1 + MO wnt5b |
| WT | 1.8E-04 | 0.70 | 6.0E-14 |
| MO mib1 |  | 4E-03 | 9.7E-04 |
| MO wnt5b |  |  | 5.3E-14 |

**Figure 5B: Axis extension angle in *mib1^tfi91^ WT siblings* + MO wnt5b**

|  | *Mean value* | *Standard deviation* | *Number of embryos* |
| --- | --- | --- | --- |
| *mib1[WT siblings]* | 189.3 | 10.4 | 133 |
| *mib1[WT siblings]*  *+ MO wnt5b* | 185.0 | 11.3 | 122 |
|  | | | |
| *Wilcoxon test:* | | | |
| p = 8.5E-03 |  |  |  |

**Figure 5C; Axis extension angle in *mib1^tfi91^ homozygous mutants* + MO wnt5b**

|  | *Mean value* | *Standard deviation* | *Number of embryos* |
| --- | --- | --- | --- |
| *mib1[tfi91/tfi91]* | 185.5 | 9.3 | 39 |
| *mib1[tfi91/tfi91]*  *+ MO wnt5b* | 180.0 | 8.1 | 28 |
|  | | | |
| *Wilcoxon test:* | | | |
| p = 0.03 |  |  |  |

**Note with respect to the statistical analyses performed in Figure 5:**

To account for the highly different sizes between WT sibling and homozygous mutant populations encountered in the experiment displayed in Figure 5B,C, statistics are performed by pairwise comparison rather than using multiple comparisons as used for the data of Figure 5A. Note that the use of similar pairwise comparisons by Wilcoxon tests for the data of Figure 5A would also confirm the conclusions already drawn from One way Anova, i.e. that MO wnt5b does not affect CE in WT controls (p=0.23) but enhances the CE defects of mib1 morphants (p=3.5E-04).
